# The metabolic-epigenetic landscape of aging: interplay between histone acetylation, lactylation, and glycation

**DOI:** 10.3389/fragi.2026.1854915

**Published:** 2026-06-03

**Authors:** Olena Bolgova, Inna Shypilova, Volodymyr Mavrych

**Affiliations:** 1 College of Medicine, Alfaisal University, Riyadh, Saudi Arabia; 2 School of Medicine, St. Matthew’s University, George Town, Cayman Islands

**Keywords:** aging, cellular senescence, epigenetics, histone acetylation, histone glycation, histone lactylation, metabolic memory, methylglyoxal

## Abstract

**Background:**

The aging epigenome is shaped by three mechanistically distinct histone post-translational modifications—acetylation, lactylation, and glycation—each driven by a different metabolic flux: mitochondrial oxidative phosphorylation, glycolytic lactate production, and reactive carbonyl stress, respectively. Understanding their interplay is central to a molecular physiology of epigenetic aging.

**Scope:**

This mini review synthesizes current evidence on the mechanisms of histone acetylation, lactylation, and glycation in aging; their crosstalk and convergence on shared regulatory nodes; and their modulation by environmental, nutritional, and behavioral factors. Key controversies and research gaps are critically appraised.

**Key Findings:**

NAD + decline in aging disables the sirtuin deacetylase family, dysregulating the histone acetylation landscape and impairing autophagy, mitochondrial biogenesis, and DNA repair. Histone lactylation, written by p300 at H3K18 and related lysine residues, is context-dependent: physiological pulses during exercise and sleep are adaptive, while chronic accumulation in diabetic microglia drives neuroinflammation via TLR4/NF-κB, and excess in tumor cells enables senescence bypass. Histone glycation by methylglyoxal irreversibly displaces regulatory marks and inactivates sirtuin proteins; pharmacological induction of glyoxalase I and glycation-lowering interventions reduce this burden and extend healthspan. These three axes may converge on a unified metabolic-epigenetic collapse that we propose constitutes the cellular basis of an ‘aging’ metabolic memory.

**Controversies and Gaps:**

Lactylation erasers remain uncharacterized; the pro-versus anti-senescence duality of H3K18la is unresolved; and genome-wide histone glycation mapping in human tissues is absent.

**Conclusion:**

Combinatorial interventions targeting NAD + restoration, modulation of lactylation, and reduction of carbonyl stress offer the most evidence-based approach to slowing metabolic-epigenetic aging.

## 1 Introduction

The chromatin of aging cells is not a passive record of molecular damage accumulated over time. It is an actively regulated information system whose state reflects (and reinforces) the metabolic condition of the cell. Post-translational modifications of histone proteins at specific lysine and arginine residues alter chromatin accessibility, recruit or repel transcription factors and remodeling complexes, and ultimately determine which genes are expressed in any given cellular context. Among the growing catalog of histone modifications, three have emerged as particularly central to the metabolic-epigenetic biology of aging: acetylation, lactylation, and glycation.

Histone acetylation, the addition of an acetyl group from acetyl-CoA to lysine ε-amino groups by lysine acetyltransferases (KATs), removed by histone deacetylases (HDACs), has been studied for decades as a marker of transcriptionally active chromatin and a readout of mitochondrial oxidative metabolism and NAD^+^ availability (Ji, 2025; Abdulkhaliq et al., 2025). Histone lactylation, identified more recently as a modification driven by lactate-derived lactyl-CoA, directly links glycolytic activity to gene regulation; lactate plays dual roles in the nervous system with functions determined by concentration and distinct signaling pathways, and lactylation has been implicated in neuroinflammation, cancer, and musculoskeletal disease (Ablitip et al., 2026; Wang et al., 2026; Xie et al., 2025; Zhang et al., 2025; Ran et al., 2026). Histone glycation, produced non-enzymatically by MGO and other reactive carbonyl species, is a largely irreversible modification that accumulates with age and metabolic disease, disrupting cellular signaling, established regulatory marks, and chromatin architecture (Kavitha et al., 2025; Wu et al., 2025).

These three axes are united by a common logic: each is a direct chemical consequence of a distinct metabolic flux (oxidative phosphorylation, glycolysis, and carbonyl stress, respectively), and each feeds back to alter gene expression in ways that either maintain homeostasis or accelerate aging. Understanding their interplay, the environmental factors that perturb their balance, and the controversies surrounding their mechanisms and therapeutic relevance is essential for developing a coherent molecular physiology of epigenetic aging.

## 2 Materials and methods

A comprehensive narrative review was conducted through systematic searches of PubMed and Scopus from inception through April 2026. The full search string was:

(((“histone acetylation” [MeSH] OR “histone acetylation” [tiab] OR “histone acetyltransferases” [MeSH] OR HAT [tiab] OR “histone deacetylases” [MeSH] OR HDAC*[tiab] OR “sirtuins” [MeSH] OR SIRT*[tiab] OR “acetyl-CoA” [tiab] OR “acetyl coenzyme A” [tiab] OR “lysine acetylation” [tiab] OR “H3K9ac” [tiab] OR “H3K27ac” [tiab] OR “H4K16ac” [tiab] OR “N-acetyltransferase” [tiab]) OR (“lactylation” [tiab] OR “histone lactylation” [tiab] OR “lysine lactylation” [tiab] OR “protein lactylation” [tiab] OR “Kla” [tiab] OR “lactate metabolism” [tiab] OR “lactate signaling” [tiab] OR “lactoyl-CoA” [tiab] OR “H3K18la” [tiab] OR “lactate-derived” [tiab] OR “Warburg effect” [tiab] OR “metabolic reprogramming” [tiab]) OR (“glycation” [MeSH] OR “glycation” [tiab] OR “advanced glycation end products” [MeSH] OR “AGEs” [tiab] OR “Maillard reaction” [tiab] OR “glycated proteins” [tiab] OR “methylglyoxal” [tiab] OR “glyoxal” [tiab] OR “RAGE” [tiab] OR “receptor for advanced glycation” [tiab] OR “carboxymethyllysine” [tiab] OR “CML” [tiab] OR “glucosepane” [tiab] OR “fructosamine” [tiab] OR “protein crosslinking” [tiab] OR “non-enzymatic glycosylation” [tiab] OR “histone glycation” [tiab])) AND ((“aging” [MeSH] OR “aging” [tiab] OR “ageing” [tiab] OR “longevity” [MeSH] OR “longevity” [tiab] OR “lifespan” [tiab] OR “healthspan” [tiab] OR “senescence” [MeSH] OR “cellular senescence” [tiab] OR “biological aging” [tiab] OR “epigenetic age” [tiab] OR “epigenetic clock” [tiab] OR “DNA methylation age” [tiab] OR “Horvath clock” [tiab] OR “GrimAge” [tiab] OR “PhenoAge” [tiab] OR “age-related” [tiab] OR “geroscience” [tiab] OR “hallmarks of aging” [tiab]) OR (“age-related diseases” [MeSH] OR “neurodegeneration” [tiab] OR “Alzheimer” [tiab] OR “Parkinson” [tiab] OR “cardiovascular aging” [tiab] OR “metabolic syndrome” [MeSH] OR “type 2 diabetes” [tiab] OR “sarcopenia” [tiab] OR “frailty” [tiab] OR “inflammaging” [tiab] OR “immunosenescence” [tiab] OR “vascular aging” [tiab] OR “cognitive decline” [tiab]))).

Eligibility criteria included peer-reviewed primary studies reporting mechanistic, preclinical, or clinical data in English. Peer-reviewed articles, clinical trials, and preclinical studies were evaluated. The 27 primary sources were selected based on relevance, methodological rigor, and contribution to the evidence base across the three epigenetic axes examined (histone acetylation, lactylation, and glycation) with priority given to studies published between 2022 and 2026 to reflect the most recent advances in the field.

## 3 Three axes of metabolic-epigenetic aging: mechanisms and therapeutic implications

### 3.1 Histone acetylation and the sirtuin-NAD^+^ axis

The cellular acetylation landscape is governed primarily by the balance between KAT-mediated deposition and HDAC-mediated removal of acetyl marks. Of the eighteen human HDACs, the seven sirtuins (SIRT1–7) are uniquely metabolic: their deacetylase activity requires NAD^+^ as an obligate co-substrate, with NAD^+^ serving as a substrate for sirtuins, PARP, and CD38, and its progressive decline in aging—through increased PARP- and CD38-mediated consumption and limited NAMPT-driven replenishment—translating directly into sirtuin hypoactivity and global dysregulation of the acetylation landscape (Ji, 2025; Wagner et al., 2022).

SIRT1 is the most extensively characterized sirtuin in the context of aging. It deacetylates both histone substrates in the nucleus and non-histone proteins in the cytosol, and its decline with age contributes to the onset and progression of multiple age-related diseases, while its activation mitigates mitochondrial dysfunction, oxidative stress, and cellular senescence (Abdulkhaliq et al., 2025). The AMPK/SIRT1/PGC-1α axis constitutes a central positive feedback loop in which AMPK activation elevates NAD^+^, activating SIRT1 to deacetylate PGC-1α, which drives mitochondrial biogenesis and function, further reinforcing SIRT1 activity; dysfunction in this cascade is implicated across neurodegenerative, metabolic, and cardiovascular diseases (Chen et al., 2025). SIRT1 activation by resveratrol delays stem cell senescence through SIRT1-dependent mitochondrial quality control, supporting the translational relevance of targeting this axis (Li Z. et al., 2025).

SIRT3, the primary mitochondrial NAD^+^-dependent deacetylase, maintains mitochondrial protein homeostasis through deacetylation and activation of SOD2 and IDH2, optimizes metabolic homeostasis, enhances mitophagy, and mediates cardiovascular protection by modulating fibrotic signaling and nitric oxide biosynthesis; its decline with aging impairs these functions across multiple tissues (Yang J. et al., 2025; You and Wang, 2025). In granulosa cells, AGE-induced senescence proceeds through SIRT3 suppression of mitophagy: SIRT3 overexpression restores mitochondrial function and recovers steroidogenesis (Li et al., 2026). In skeletal myoblasts, the flavonoid DMF acts as a SIRT3 activator, enhancing SOD2 deacetylation and activation, reducing mitochondrial ROS, and suppressing NF-κB-driven inflammation, with all its anti-senescent effects abolished upon SIRT3 knockdown (Li B. et al., 2025). SIRT6 in the nucleus regulates tryptophan catabolism through control of TDO2 and AANAT gene expression: its decline in aging elevates neurotoxic kynurenic pathway metabolites at the expense of serotonin and melatonin production, impairing sleep, neuromotor function, and cognitive resilience; since tryptophan byproducts are involved in NAD^+^ biosynthesis, this diversion of tryptophan flux may also reduce the availability of NAD^+^ precursors generated through the kynurenine route (Kaluski-Kopatch et al., 2025).

Class I HDACs (HDAC1, 2, 3, 8) and HDAC6 are the zinc-dependent counterparts to sirtuins and exhibit distinct dysregulation in age-related diseases. In cardiac aging, alterations in energy metabolism contribute to dysfunction, with both mitochondrial hyperacetylation and an acetyltransferase/deacetylase imbalance playing central roles. Caloric restriction and HDAC inhibitors are examined as potential therapeutic approaches (Hase and Banerjee, 2025). HDAC2 dysregulation contributes to AD pathology through synaptic dysfunction, amyloid-β accumulation, and tau tangle formation, and its selective modulation is proposed as a therapeutic avenue (Asadi et al., 2025). Pan- and isoform-selective HDAC inhibitors have demonstrated preclinical efficacy against multiple neurodegenerative diseases, with HDAC6-selective inhibitors showing promising preclinical efficacy in AD, PD, and HD pathology, and BBB-penetrant and PROTAC-based scaffolds prioritized for clinical translation (Pawar et al., 2026; Joshi et al., 2025). In type 2 diabetes, HDAC1, 2, and 3 expression is elevated in obese individuals relative to lean controls, and metformin treatment upregulates HDAC1, 3, and 8, suggesting that part of metformin’s benefit involves modulation of class I HDACs (Izadi et al., 2025). HAT/HDAC balance additionally controls oligodendrocyte precursor cell differentiation into myelinating oligodendrocytes during remyelination; disruptions in reversible acetylation contribute to neurodegenerative impairments and accelerated ageing in the CNS (Dominicis et al., 2025). The rapidly evolving sirtuin modulator landscape (encompassing activators and isoform-selective inhibitors, with PROTAC technology enabling selective degradation of specific isoforms) holds potential for precision therapeutic targeting across aging, neurodegeneration, and metabolic disease (Fabbrizi et al., 2026).

### 3.2 Histone lactylation: the glycolytic epigenome and its dual roles

The recognition that L-lactate can enzymatically modify histone lysine residues through lactyl-CoA to produce a distinct epigenetic mark has fundamentally expanded the metabolic-epigenetic paradigm. Key sites include H3K18la, H3K9la, H4K5la, and H4K8la. The acetyltransferase p300 has been identified as a lactylation writer: TRPM7 deficiency in vascular endothelial cells reduces lactate production and inhibits p300, leading to decreased H3K18 lactylation, increased p21 expression, and premature vascular aging, directly demonstrating that p300-mediated H3K18la is required to suppress senescence gene programs in the endothelium (Wang et al., 2025). H3K18la critically competes with the acetylation mark H3K18ac at the same lysine residue, meaning the ratio of lactyl-CoA to acetyl-CoA (a direct readout of glycolytic versus oxidative metabolic balance) mechanistically determines transcriptional output at H3K18-regulated loci (Ablitip et al., 2026; Wang et al., 2025; Yang Y. et al., 2025).

In the nervous system, lactate’s roles extend well beyond energy provision. The astrocyte-neuron lactate shuttle (ANLS) distributes lactate to neurons, where it acts as a signaling molecule influencing neuroplasticity via NMDA receptors, modulating neuroinflammation via the HCAR1 receptor, and regulating gene expression through histone lactylation (Ablitip et al., 2026). During slow-wave sleep, brain lactate naturally increases to support memory consolidation and glymphatic clearance, whereas exercise-induced transient lactate surges prime the brain’s metabolic infrastructure via these pathways, suggesting that physiological lactate pulses are adaptive (Ablitip et al., 2026). Lactate is additionally involved in neuroinflammation and neurodegenerative disorders, including Alzheimer’s and Parkinson’s disease, with lactylation status influencing disease progression through its effects on glial cells and neurons (Cao et al., 2025). Glycolytic reprogramming of activated microglia is a key driver of this process: the resulting elevation in lactate promotes histone lactylation, which significantly alters gene expression and sustains chronic neuroinflammation (Wang et al., 2026). In the diabetic brain specifically, H3K18la directly stimulates NF-κB signaling by increasing binding at the TLR4 promoter, thereby promoting M1 microglial polarization and contributing to diabetes-associated cognitive impairment (Yang Y. et al., 2025).

In musculoskeletal tissues, aberrant lactylation has been implicated in the disruption of osteogenic-osteoclastic balance, extracellular matrix homeostasis, and regenerative capacity, linking cellular metabolic stress to pathological tissue remodeling across osteoporosis, osteoarthritis, and sarcopenia (Xie et al., 2025). Bioinformatics analysis of sarcopenic muscle has identified MDH1 (a gene involved in both glycolysis and lactylation) as a hub gene predominantly expressed in myonuclei and NKT cells, with the strongest association with immune infiltration observed in regulatory T cells, and hsa-miR-1263 identified as its upstream regulatory microRNA (Luo et al., 2025). The therapeutic implication, that LDH inhibitors reducing pathological lactate production could modulate the SASP and fibrotic extracellular matrix deposition, is gaining mechanistic support (Luo et al., 2026).

A significant controversy concerns whether lactylation is primarily a protective adaptive signal or a pathological driver of age-associated disease. In NSCLC, lactate-derived H3K18la activates KRT19 transcription by directly binding its promoter; KRT19 in turn blocks p53-mediated p21 transcription and facilitates p21 ubiquitination via MYH9 interaction, enabling tumor cells to bypass senescence (Zhang et al., 2025). This anti-senescence function of H3K18la in cancer stands in direct contrast to its pro-senescence role demonstrated in the TRPM7-deficient endothelial model, where reduced H3K18la promotes p21-driven premature vascular aging (Wang et al., 2025). The same mark thus suppresses senescence in tumor cells while its loss accelerates senescence in endothelial cells, underscoring that the functional consequence of lactylation depends critically on cellular context, downstream readers, and co-occurring epigenetic states. These opposing outcomes most likely reflect differences in cell-type-specific reader proteins, the competitive balance between H3K18 lactylation and acetylation, and the loci involved—none of which can be resolved without genome-wide lactylation maps. This ambiguity is therapeutically consequential: suppressing H3K18la in one tissue could harm another. Lactylation erasers remain entirely uncharacterized, fundamentally limiting understanding of reversibility and therapeutic targeting (Wang et al., 2026; Xie et al., 2025).

### 3.3 Histone glycation: carbonyl stress and irreversible chromatin damage

In contrast to the enzymatically regulated modifications above, histone glycation is predominantly non-enzymatic and, in many cases, irreversible. MGO, generated by spontaneous degradation of triose phosphate intermediates during glycolysis, reacts with lysine ε-amino and arginine guanidino groups on histone tails to form Schiff bases and Amadori products that rearrange into stable AGEs. Because glycation target sites on histone tails overlap with acetylation, methylation, and ubiquitination residues, glycation displaces established regulatory marks and chemically occludes the binding of modifying enzymes, creating transcriptionally inert chromatin regions that expand with age (Kavitha et al., 2025; Wu et al., 2025).

Pharmacological induction of glyoxalase I (Glo1) through Nrf2 activation by the trans-resveratrol and hesperetin combination (tRES + HESP) counters MGO accumulation and reduces senescence markers, inflammation, and glycolytic overload-related gene expression in human endothelial and hepatic cell models; the combination was also previously demonstrated in a clinical trial to correct insulin resistance and reduce inflammation in overweight subjects (Xue et al., 2025; note: corresponding authors hold a patent on this formulation). Both dietary and endogenous AGEs sustain pro-inflammatory programs and oxidative stress by activating NF-κB and JNK signaling through RAGE, which further accelerates AGE formation in a self-amplifying loop (Kavitha et al., 2025); RAGE additionally activates MAPK pathways and has been positioned as a central therapeutic target across diabetes and Alzheimer’s disease, with dietary bioactive compounds including polyphenols, polysaccharides, and terpenoids representing tractable intervention strategies (Wu et al., 2025). In vascular endothelial cells, MGO drives senescence through EIF2AK2/PKR dimerization and integrated stress response activation, upregulating p16, p21, and p53 while suppressing Bcl-2; berberine attenuates this pathway through selective EIF2AK2 inhibition (Chen et al., 2026). In ovarian granulosa cells, AGEs induce senescence by disrupting mitochondrial function and suppressing PINK1/Parkin-mediated mitophagy, with SIRT3 overexpression restoring mitophagy and steroidogenesis, directly linking the glycation and sirtuin axes (Li et al., 2026). In skeletal muscle, MGO administration in mice suppresses MyoD and myogenin expression, upregulates MuRF1 and Atrogin-1, and increases fibrosis; aerobic exercise prevents these effects; in parallel human data, skin autofluorescence and CML levels correlated positively with AGE burden and negatively with grip strength and functional performance in adults aged 65 and older (Lee M. J. et al., 2025). Independent evidence further supports the beneficial effects of exercise on AGE-related muscle deterioration (Wu et al., 2026). The glycation-lowering supplement Gly-Low reduced MGO-derived AGEs, improved insulin sensitivity while preserving muscle mass, extended lifespan in mice, and, as a late-life intervention, slowed hypothalamic aging signatures and improved motor coordination (Wimer et al., 2025) (Table 1).

**TABLE 1:** Comparison of histone acetylation, lactylation, and glycation in the context of aging.

Feature	Histone acetylation	Histone lactylation	Histone glycation
Metabolic driver	Acetyl-CoA (mitochondrial OXPHOS, NAD + cycle)	Lactyl-CoA (glycolytic lactate)	Methylglyoxal and reactive carbonyls (triose phosphate degradation)
Enzymatic/non-enzymatic	Enzymatic (KATs write; HDACs/sirtuins erase)	Enzymatic (p300 confirmed as writer (Wang et al., 2025); eraser uncharacterized)	Non-enzymatic; largely irreversible AGE formation
Age-related change	Decreased at homeostatic loci; increased at inflammatory loci	Increased in glycolytic/diabetic states; reduced H3K18la accelerates vascular senescence (Wang et al., 2025)	Progressively accumulates; counteracted by glyoxalase system (Xue et al., 2025)
Primary functional consequence	Chromatin opening, gene activation; loss disrupts autophagy, DNA repair, mitochondrial biogenesis	Context-dependent: physiological lactylation is adaptive (Ablitip et al., 2026); excess drives neuroinflammation (Wang et al., 2026; Yang et al., 2025b); loss accelerates vascular senescence (Wang et al., 2025)	Displacement of regulatory marks; sirtuin protein inactivation; chromatin rigidity
Key disease associations	Neurodegeneration, T2DM, sarcopenia, cardiovascular aging	Alzheimer’s/Parkinson’s disease, diabetes-associated cognitive impairment, NSCLC, sarcopenia, vascular aging	Vascular senescence, ovarian aging, muscle atrophy, diabetic neurodegeneration
Therapeutic strategies	NAD + precursors (NMN/NR, nicotinamide), HDAC inhibitors, sirtuin activators	LDH inhibitors (Luo et al., 2026), p300 modulation, glycolysis modulators	Glo1 inducers (tRES + HESP) (Xue et al., 2025), MGO-lowering supplements (Gly-Low) (Wimer et al., 2025), RAGE antagonists (Wu et al., 2025)
Key references	Ji (2025), Abdulkhaliq et al. (2025), Pawar et al. (2026)	Wang et al. (2026), Zhang et al. (2025), Wang et al. (2025), Yang et al. (2025b)	Xue et al. (2025), Chen et al. (2026), Wimer et al. (2025)

## 4 Discussion

**FIGURE 1:**
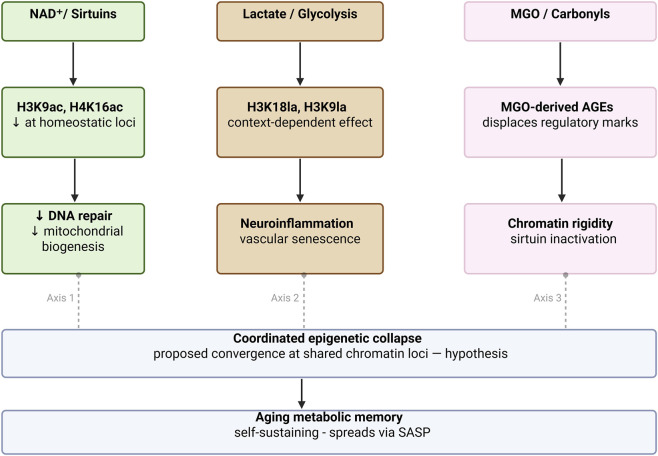
Three-axis metabolic-epigenetic convergence model of aging. Each column represents one axis: acetylation (NAD^+^/sirtuin, green), lactylation (lactate/glycolysis, brown), and glycation (MGO/carbonyls, pink). The downstream epigenetic marks and functional consequences of each axis are shown vertically within each column. Dashed lines connecting the three axes at the base indicate the proposed, but not yet directly demonstrated, convergence at shared chromatin loci (hypothesis). The collective outcome, aging metabolic memory, is represented as a self-sustaining epigenetic attractor propagated intercellularly via SASP components. Citation: Created in BioRender. Shypilova et al. (2026); https://BioRender.com/70mlhk9.

The evidence synthesized in this review supports a unified metabolic-epigenetic model of aging in which three histone modification axes (acetylation, lactylation, and glycation) function as an integrated sensory network translating the cell’s metabolic state into durable chromatin-encoded programs (Figure 1).

Their convergence is not coincidental. The age-associated shift from mitochondrial oxidative phosphorylation toward aerobic glycolysis (driven by mitochondrial dysfunction, NAD^+^ depletion, and accumulated oxidative damage) simultaneously reduces acetyl-CoA availability and NAD^+^-dependent sirtuin activity (impairing acetylation control), increases lactyl-CoA production and pathological H3K18la accumulation (driving aberrant lactylation), and raises MGO while glyoxalase-mediated detoxification becomes insufficient (escalating histone glycation). The result is a coordinated epigenetic collapse that reinforces the metabolic state that caused it - a self-sustaining attractor that, we hypothesize, constitutes the cellular basis of the ‘aging metabolic memory’ concept, in which senescent epigenetic imprints are self-perpetuating and spread intercellularly through SASP components and extracellular vesicles (Zhao, 2026).

Several important controversies complicate this picture. First, the identity of lactylation erasers remains entirely uncharacterized: while p300 is confirmed as a writer (Wang et al., 2025), the enzymes that reverse lactylation are unknown, fundamentally limiting understanding of this mark’s reversibility and constraining rational therapeutic targeting (Wang et al., 2026; Xie et al., 2025). Second, the functional duality of H3K18la—suppressing senescence in endothelial cells where its reduction accelerates aging (Wang et al., 2025), yet driving pro-inflammatory microglial states and enabling senescence bypass in cancer where it accumulates (Wang et al., 2026; Zhang et al., 2025) — constitutes a genuine biological paradox that must be resolved before lactylation-modulating therapies can be safely developed, as interventions that reduce lactylation to dampen neuroinflammation could theoretically accelerate vascular senescence. Third, while NAD^+^ supplementation holds therapeutic promise, the precise *in vivo* NAD^+^ metabolism, tissue-specific bioavailability, and long-term clinical safety of precursor strategies remain under investigation and lack complete clinical validation (Wagner et al., 2022; Fabbrizi et al., 2026).

Critical research gaps persist across all three axes. For histone glycation, comprehensive genome-wide mapping of MGO-derived histone adducts at single-locus resolution in aged human tissues is lacking; existing evidence primarily derives from mass spectrometry of bulk histone preparations or cell culture models (Kavitha et al., 2025; Wu et al., 2025). The extent to which histone glycation is reversible remains largely unknown, which is fundamental to determining whether anti-glycation strategies can reverse established epigenetic damage or only prevent further accumulation. For lactylation, tissue-specific modification patterns, circadian dynamics, and the identity of downstream reader proteins have not been systematically characterized (Wang et al., 2026; Xie et al., 2025). For the acetylation axis, whether declining sirtuin activity at specific loci is a cause or consequence of senescence remains unresolved for many targets (Ji, 2025; Abdulkhaliq et al., 2025).

The strongest translational evidence supports combinatorial strategies (Table 2). Exercise activates SIRT1 through NAD^+^/AMPK signaling, activates Nrf2-linked antioxidant programs, and reduces carbonyl stress (Hoseini et al., 2025; Lee M. J. et al., 2025; Lee J. M. et al., 2025); integrative lifestyle strategies combining exercise, nutrition, and bioactive compounds have shown synergistic potential in counteracting age-related hormonal and metabolic decline, further supporting SIRT1/AMPK axis restoration (Shypilova et al., 2026); time-restricted feeding restores the AMPK/SIRT1/PGC-1α axis and improves endothelial epigenetic health (Milan et al., 2026); DHA combined with exercise specifically and synergistically preserves telomere integrity and SIRT3/FOXO3 antioxidant programs (Gámez-Macías et al., 2026); gut microbiota-derived metabolites modulate host epigenetic circuits and support SIRT1/AMPK activity at longevity-associated loci (Protachevicz et al., 2026); and NAD^+^ precursors restore the sirtuin substrate pool depleted in aging (Ji, 2025; Nowacka et al., 2025). RAGE-targeted dietary interventions offer a preventive nutritional approach aligned with the WHO concept of preventive aging (Wu et al., 2025), and natural dietary strategies targeting estrogen optimization in aging women similarly align with this preventive framework, addressing the hormonal dimension of metabolic-epigenetic aging (Bolgova et al., 2025). Pharmacologically, sirtuin activators, isoform-selective HDAC inhibitors, LDH inhibitors, and Glo1 inducers converge on the same metabolic-epigenetic network and provide a basis for rational combination therapy (Pawar et al., 2026; Fabbrizi et al., 2026; Luo et al., 2026). Complementing these pharmacological approaches, naturally derived bioactive compounds and therapeutic peptides represent an emerging class of interventions targeting the same metabolic-epigenetic network, with growing evidence supporting their role in healthy aging (Clemens et al., 2026; Mavrych et al., 2026).

**TABLE 2:** Environmental and nutritional modulators of the metabolic-epigenetic axes of aging.

Modulator	Primary epigenetic effects	Molecular mechanism	Key references
Aerobic exercise	Activates SIRT1 via NAD+/AMPK; reduces AGE burden in muscle; activates Nrf2-linked antioxidant programs	NAD+/AMPK elevation upregulates SIRT1 and reduces mTOR; Nrf2-linked antioxidant gene expression; transient adaptive lactylation	Lee et al. (2025a), Hoseini et al. (2025), Lee et al. (2025b)
Caloric restriction/time-restricted feeding	Restores SIRT1/AMPK/PGC-1α axis; suppresses mTOR; improves endothelial epigenetic health	AMPK and SIRT1 activation; metabolic switch toward ketone utilization; mitochondrial and vascular repair	Chen et al. (2025), Milan et al. (2026)
High-glycemic diet/obesity	Promotes AGE accumulation; increases class I HDAC expression; activates RAGE/NF-κB/JNK	MGO overproduction; NAD+/NAMPT suppression; NF-κB and JNK activation via AGE-RAGE	Kavitha et al. (2025), Izadi et al. (2025), Wimer et al. (2025)
Omega-3 fatty acids (DHA) combined with exercise	Preserves liver telomere integrity; maintains SIRT3, FOXO3, SOD1, catalase gene expression	Antioxidant gene induction; combined mitochondrial protection; effective only when both interventions combined	Gámez-Macías et al. (2026)
Gut microbiota SCFAs	Modulate host epigenetic circuits via substrates for writer/eraser enzymes; support SIRT1/AMPK; attenuate NF-κB/COX-2	Microbiota-derived metabolites as epigenetic enzyme substrates; lower LPS in centenarians supports enhanced SIRT1 activity via the LPS–SIRT1 axis	Protachevicz et al. (2026)
NAD + precursors (nicotinamide/NMN)	Restores sirtuin activity; supports autophagy and stem cell maintenance; improves mitochondrial function	NAMPT cofactor provision; sirtuin reactivation; NAD + homeostasis restoration	Ji (2025), Wagner et al. (2022), Nowacka et al. (2025)
Glo1 inducers (tRES + HESP, Gly-Low)	Reduces histone glycation; decreases senescence markers; improves metabolic parameters	Nrf2-driven Glo1 induction; MGO scavenging; AGE-RAGE axis suppression	Xue et al. (2025), Wimer et al. (2025)
Natural compounds (resveratrol, DMF, astaxanthin, berberine)	SIRT1/3 activation; EIF2AK2 inhibition; NRF2-SIRT3 axis induction	SIRT1-dependent mitochondrial quality control; SIRT3-SOD2 activation; MGO-PKR pathway blockade	Li et al. (2025a), Li et al. (2025b), Chen et al. (2026), Clemens et al. (2026)

Looking forward, the most transformative advance will come from integrating single-cell multi-omic profiling—simultaneously capturing histone modification landscapes, chromatin accessibility, and metabolomics—with validated epigenetic aging clocks across large human longitudinal cohorts, enabling causal inference about which modification changes precede functional decline, identifying tissue-specific patterns, and establishing biomarkers capable of measuring the pace of metabolic-epigenetic aging and response to intervention. The metabolic-epigenetic landscape of aging is becoming not only legible but, with growing mechanistic precision, increasingly rewritable.

## 5 Conclusion

The metabolic-epigenetic landscape of aging is defined by the convergent dysregulation of three histone modification axes—acetylation, lactylation, and glycation—each mechanistically coupled to a distinct metabolic flux and collectively constituting a self-reinforcing epigenetic collapse that encodes and perpetuates the aging state at the chromatin level. NAD^+^ depletion silences the sirtuin-dependent acetylation machinery, disrupting autophagy, mitochondrial biogenesis, and DNA repair; context-dependent shifts in lactylation drive pathological neuroinflammation and vascular senescence while paradoxically enabling senescence bypass in malignant cells; and the progressive accumulation of MGO-derived histone glycation irreversibly occludes regulatory sites, inactivates sirtuin proteins, and rigidifies chromatin in ways that current interventions can attenuate but not yet fully reverse. The most evidence-based path forward lies in combinatorial strategies that simultaneously target all three axes—restoring the sirtuin substrate pool through NAD^+^ precursors, modulating glycolytic flux and lactylation dynamics through exercise and LDH inhibition, and reducing carbonyl stress through Glo1 inducers and dietary AGE restriction—while closing the critical gaps that remain: characterizing lactylation erasers, resolving the pro-versus anti-senescence duality of H3K18la, and generating genome-wide histone glycation maps in aged human tissues. As single-cell multi-omic technologies and longitudinal epigenetic aging clocks mature, the metabolic-epigenetic network described here will become not only a mechanistic framework for understanding aging but a precision target for rewriting it.

## Glossary

AGEs: advanced glycation end products
AMPK: AMP-activated protein kinase
AANAT: arylalkylamine N-acetyltransferase
ANLS: astrocyte-neuron lactate shuttle
Atrogin-1: muscle atrophy F-box protein
Bcl-2: B cell lymphoma 2 protein
BER: base excision repair
CML: carboxymethyllysine
CNS: central nervous system
COX-2: cyclooxygenase-2
DMF: 6,4′-dihydroxy-7-methoxyflavanone
EIF2AK2: eukaryotic initiation factor 2 alpha kinase 2 (also known as PKR)
FOXO3: forkhead box O3 transcription factor
Glo1: glyoxalase I
H3K18ac: histone H3 lysine 18 acetylation
H3K18la: histone H3 lysine 18 lactylation
H3K9la: histone H3 lysine 9 lactylation
H4K5la: histone H4 lysine 5 lactylation
H4K8la: histone H4 lysine 8 lactylation
HAT: histone acetyltransferase
HCAR1: hydroxycarboxylic acid receptor 1
HDAC: histone deacetylase
HESP: hesperetin
IDH2: isocitrate dehydrogenase 2
JNK: c-Jun N-terminal kinase
KAT: lysine acetyltransferase
KRT19: keratin 19
Kla: lysine lactylation
LDH: lactate dehydrogenase
LPS: lipopolysaccharide
MAPK: mitogen-activated protein kinase
MDH1: malate dehydrogenase 1
MGO: methylglyoxal
mTOR: mechanistic target of rapamycin
MuRF1: muscle RING-finger protein 1
MyoD: myoblast determination protein 1
MYH9: myosin heavy chain 9
**NAD** ^**+**^: nicotinamide adenine dinucleotide
NAMPT: nicotinamide phosphoribosyltransferase
NF-κB: nuclear factor kappa-light-chain-enhancer of activated B cells
NMN: nicotinamide mononucleotide
NR: nicotinamide riboside
Nrf2: nuclear factor erythroid 2-related factor 2
NSCLC: non-small cell lung cancer
OXPHOS: oxidative phosphorylation
PARP: poly(ADP-ribose) polymerase
PGC-1α: peroxisome proliferator-activated receptor gamma coactivator 1-alpha
PINK1: PTEN-induced kinase 1
PKR: protein kinase R (see EIF2AK2)
PROTAC: proteolysis-targeting chimera
RAGE: receptor for advanced glycation end products
ROS: reactive oxygen species
SASP: senescence-associated secretory phenotype
SCFAs: short-chain fatty acids
SIRT1–7: sirtuin 1 through 7
SOD2: superoxide dismutase 2
T2DM: type 2 diabetes mellitus
TDO2: tryptophan 2,3-dioxygenase
TLR4: toll-like receptor 4
tRES: trans-resveratrol
TRPM7: transient receptor potential melastatin 7
